# ELISA Test for the Serological Detection of *Scedosporium*/*Lomentospora* in Cystic Fibrosis Patients

**DOI:** 10.3389/fcimb.2020.602089

**Published:** 2020-11-26

**Authors:** Leire Martin-Souto, Idoia Buldain, Maialen Areitio, Leire Aparicio-Fernandez, Aitziber Antoran, Jean-Philippe Bouchara, Maria Teresa Martin-Gomez, Aitor Rementeria, Fernando L. Hernando, Andoni Ramirez-Garcia

**Affiliations:** ^1^Fungal and Bacterial Biomics Research Group, Department of Immunology, Microbiology and Parasitology, University of the Basque Country (UPV/EHU), Leioa, Spain; ^2^Groupe d’Etude des Interactions Hôte-Pathogène (EA 3142), SFR ICAT 4208, Institut de Biologie en Santé-IRIS, Centre Hospitalier Universitaire, Angers, France; ^3^Microbiology Department, Vall d’Hebron University Hospital, Barcelona, Spain

**Keywords:** enzyme linked immunosorbent assay, *Scedosporium*, cystic fibrosis, serodiagnosis, *Lomentospora*

## Abstract

The detection and diagnosis of the opportunistic fungi *Scedosporium* spp. and *Lomentospora prolificans* still relies mainly on low-sensitive culture-based methods. This fact is especially worrying in Cystic Fibrosis (CF) patients in whom these fungal species are frequently isolated and may increase the risk of suffering from an infection or other health problems. Therefore, with the purpose of developing a serologic detection method for *Scedosporium*/*Lomentospora*, four different *Scedosporium boydii* protein extracts (whole cell protein extract, secretome, total cell surface and conidial surface associated proteins) were studied by ELISA to select the most useful for IgG detection in sera from CF patients. The four extracts were able to discriminate the *Scedosporium*/*Lomentospora*-infected from *Aspergillus-*infected and non-infected patients. However, the whole cell protein extract was the one selected, as it was the one with the highest output in terms of protein concentration per ml of fungal culture used, and its discriminatory capacity was the best. The ELISA test developed was then assayed with 212 sera from CF patients and it showed to be able to detect *Scedosporium* spp. and *Lomentospora prolificans* with very high sensitivity and specificity, 86%–100% and 93%–99%, respectively, depending on the cut-off value chosen (four values were proposed A_450nm_= 0.5837, A_450nm_= 0.6042, A_450nm_= 0.6404, and A_450nm_= 0.7099). Thus, although more research is needed to reach a standardized method, this ELISA platform offers a rapid, low-cost and easy solution to detect these elusive fungi through minimally invasive sampling, allowing the monitoring of the humoral response to fungal presence.

## Highlights

In recent years huge efforts have been made to develop new serological techniques to improve diagnosis of fungal infections. However, most of the advances are focused on high prevalence fungal pathogens such as *Aspergillus* or *Candida*. Regarding less common fungi like *Scedosporium/Lomentospora*, which are considered emerging pathogens and are gaining clinical relevance due to the severity of the infections they cause, there are no commercial detection methods available. This manuscript describes an ELISA test developed using a whole cell protein extract from *Scedosporium boydii* that offers a great sensitivity and specificity to detect *Scedosporium/Lomentospora* in Cystic Fibrosis (CF) patients using serum samples. Compared with the commercial kits for other fungi, which usually show a 75%–96% of sensitivity, the clinical usefulness of the test developed is remarkable considering that values obtained are of 86%–100% for sensitivity and 93%–99% for specificity. These results place it in a good position as a candidate to be used as a diagnostic tool. Thus, this ELISA platform is a rapid, low-cost, and easy solution to detect *Scedosporium/Lomentospora*, allowing the monitoring of the antifungal humoral response.

## Introduction

Cystic fibrosis (CF) is the major genetic disorder among the Caucasian population (Mirtajani et al., 2017). This multisystem disease is caused by mutations in the CFTR (Cystic Fibrosis Transmembrane Conductance Regulator) gene encoding a chloride-conducting transmembrane channel, which participates in electrolytic transport and mucociliary clearance of the airways. CFTR dysfunction results in an increased viscosity of secretions and underlies an altered immune response of these patients (Rowe et al., 2005). Although several organs are adversely affected, morbimortality is essentially associated with lesions in the lungs (Castellani & Assael, 2017). The airways of CF patients are affected by the accumulation of a thick layer of sticky bronchial mucus, which acts as a culture media for microorganisms, entrapping airborne bacteria and fungal spores, immobilizing them and facilitating their growth. In this sense, these pathogens cause chronic respiratory infections, turning the lung into an inflammatory microenvironment and eventually leading to pulmonary damage (Elborn, 2016).

Even though bacteria, such as *Pseudomonas aeruginosa* or *Staphylococcus aureus*, are known to be the major causative agents of these infections, several fungal species colonize the respiratory tract of CF patients. However, while the relevance of bacteria is well known, the clinical significance of fungal recovery from respiratory secretions remains unclear (Schwarz et al., 2018). *Candida albicans*, among yeasts, and *Aspergillus fumigatus*, among filamentous fungi, are the fungal species most frequently isolated from CF respiratory samples. However, fungi from *Scedosporium* genus are increasingly reported in the CF context and currently rank second, just behind *A. fumigatus*, among the filamentous fungi colonizing CF airways (LiPuma, 2010).

Despite airway colonization by *Scedosporium* spp., or the strongly related *Lomentospora prolificans* (Ramirez-Garcia et al., 2018), it is usually well tolerated, it may lead to a true respiratory infection with variable degree of tissue invasion, fungal sensitization or allergic bronchopulmonary mycoses (Martín-Gómez, 2020). In fact, these fungal pathogens seems to be more representative during scenarios of moderate-to-severe alteration of lung function, and their presence on CF airways has been associated with a decline in Forced Expiratory Volume in 1 s (FEV1) (Soret et al., 2020). In addition, the chronic detrimental presence of these pathogens may cause fatal disseminated infections when the patient undergoes an immunosuppression period, for example after a lung transplantation (Symoens et al., 2006).

Unfortunately, lung infection or colonization by *Scedosporium*/*Lomentospora* is nowadays a diagnostic and therapeutic challenge in CF patients. Despite the many methods of *Aspergillus* detection being currently available, the detection of *Scedosporium/Lomentospora* relies upon low sensitivity culture-based traditional methods. Recently, some advances have been made on molecular diagnosis, but these methodologies are not standardized and not accessible to everyone, and serodiagnosis strategies are performed only in specialized laboratories, but these are not commercially available (Mina et al., 2017). Therefore, the precise diagnosis of these fungi is actually a significant challenge. Furthermore, the clinical features and histopathology of infected tissue samples are similar to those of aspergillosis, so confirmation data of these species may often be underestimated (Mello et al., 2019). Hence, detection and correct discrimination of *Scedosporium* infections from others is of crucial importance because the treatments may be quite different. Indeed, *Scedosporium*/*Lomentospora* species are considered intrinsically resistant to most of the currently available antifungal drugs (Pellon et al., 2018).

In this sense, to contribute to the finding of new diagnostic weapons that allow prevention, early diagnosis, and ultimately a more effective treatment, our research group analysed different protein extracts of *Scedosporium boydii* as serodiagnostic tools to detect *Scedosporium/Lomentospora* and discriminate them from other fungal pathogens relevant in the CF context. Therefore, in this study we designed and tested a customized serological assay for the detection of *Scedosporium*-specific IgG antibodies in sera from CF patients.

## Materials and Methods

### Microorganisms and Culture Conditions

The fungal strains used in this study were *Scedosporium boydii* CBS 116995, *Lomentospora prolificans* CECT 20842, *Aspergillus fumigatus* Af293 and *Candida albicans* NCPF 3153. All strains were maintained cryopreserved at -80ºC and cultured as required on Potato Dextrose Agar (PDA) (Pronadisa, Madrid, Spain).

To harvest conidiospores of *S. boydii* and *L. prolificans*, PDA plates grown at 37ºC for 7 days were washed twice with sterile saline solution (0.9% [w/v] NaCl) (SS). The suspension of conidia was filtered through sterile gauze to avoid cell debris and centrifuged. Conidia of *A. fumigatus* were collected from PDA tubes grown at 37ºC for 4 days using sterile SS-Tween20 (0.9% [w/v] NaCl, 0.02% [v/v] Tween20), and washed twice by centrifugation. Finally, *C. albicans* was grown in PDA tubes at 37ºC for 24 h, and yeast cells were gathered next day by resuspending the culture with phosphate buffered saline (PBS). The concentration of each fungal cell was adjusted as needed using a hemocytometer.

### Human Serum Sample Collection and Categorization

A collection of 212 sera from CF patients (corresponding to 102 different patients) were used in this study with the approval of the Ethics Committee from the University of the Basque Country (UPV/EHU; reference M30/2018/081).

The categorization of the sera was based on the results of mycological examination of a sputum sample collected in parallel to the sera and inoculated on Sabouraud gentamicin chloramphenicol agar, and simultaneously on Modified Thayer Martin agar or Sabouraud chloramphenicol agar supplemented with 0.5 g/L cycloheximide for the specific recovery of *Scedosporium* species from these polymicrobial samples. All plates were incubated at 37ºC for up to 15 days before to consider the sputum samples as free of fungi. According to these, three groups of sera were defined: Group Scedo+ (n = 23) consisted of sera from CF patients with positive cultures for *Scedosporium*/*Lomentospora*, Group Asp+ (n = 86), CF patients with *Aspergillus* spp. being the only filamentous fungi recovered from sputum; and group Scedo-/Asp- (n = 103) as control, consisted of sera from CF patients without any filamentous fungus recovered from samples. Sera from patients with co-infection of *Scedosporium*/*Lomentospora* and *Aspergillus* spp. were included in the group Scedo+.

In addition, five sera from each group described above were selected to evaluate the usefulness of different protein extracts for *Scedosporium*/*Lomentospora* serodiagnosis, and to study cross-reactivity with other fungal pathogens. To do that, two criteria were followed: each serum corresponded to a different patient without any coinfections.

### Fungal Protein Extracts

Four different protein extracts were obtained for this study: whole cell protein extract (Total WCP), extract of secreted proteins (Secretome), cell surface associated proteins (Total CSP) and conidial surface proteins (Conidial CSP). All extracts were obtained for *S. boydii*, but the Total WCP was also obtained for *L. prolificans, A. fumigatus*, and *C. albicans*. The resulting protein extracts were stored at -80ºC until required.

**Figure 1:**
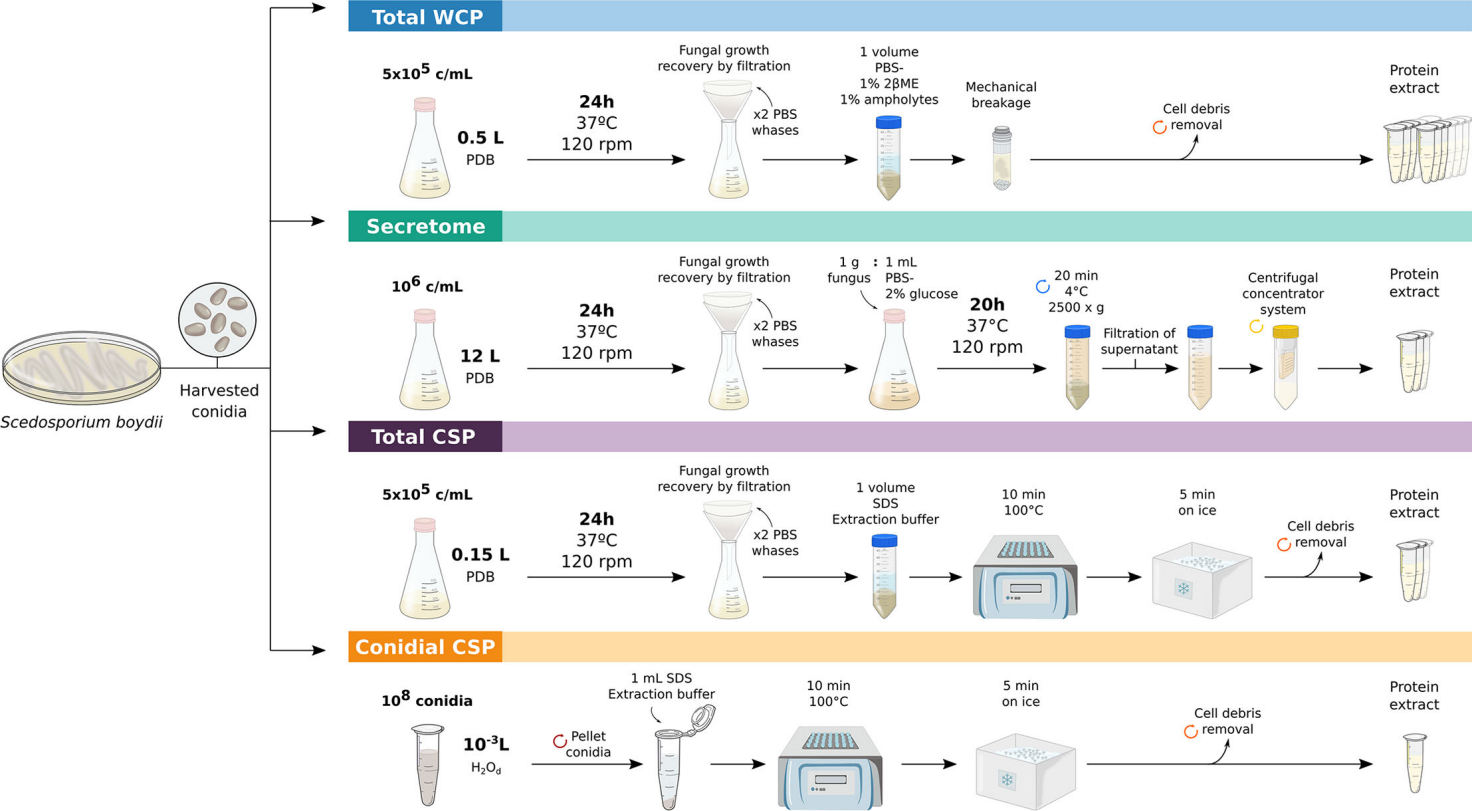
Workflows employed for obtaining *S. boydii* protein extracts. Key-steps in the obtaining of crude total extract (Total WCP), secretome, total extract of cell-surface associated proteins (Total CSP) and conidial cell-surface associated proteins (Conidial CSP) of *S. boydii*.

The extraction processes (**Figure 1**) were carried out in triplicate and the quality of the extract was verified by SDS-PAGE in 12% polyacrylamide gels, stained afterwards as previously described (Dyballa and Metzger, 2009) with Coomassie Brilliant Blue G250 (CBB), and digitalized using ImageScanner III (GE Healthcare, Chicago, IL, USA). Protein concentration was quantified using Pierce 660 nm Protein Assay Reagent (Thermo Fisher Scientific, Rockford, IL, USA). Likewise, to avoid interference from some reagents present in the extraction buffers, protein extracts were precipitated with a solution of acetone and 10% (w/v) trichloroacetic acid, and resuspended in distilled water, prior to protein concentration measurement.

#### Whole Cell Protein Extract of Conidia and Hyphae (Total WCP)

To obtain total protein extracts, 5 x 10^5^ cells/ml (conidia or yeasts) were inoculated into Potato Dextrose Broth (PDB) (Pronadisa, Madrid, Spain) cultured at 37ºC and 120 rpm for 24 h. Fungal growth was recovered by filtration and washed twice with PBS to remove traces from the medium. Fungal material was resuspended in PBS supplemented with 1% (v/v) β-mercaptoethanol and 1% (v/v) ampholytes pH 3-10 (GE Healthcare, Freiburg, Germany). Finally, cell disruption was achieved by bead-beating with glass beads for 20 min at 30 Hz using the MillMix20 (Tethnica, Slovenia), following the standardized protocol described previously (Pellon et al., 2016). Cell debris was discarded by centrifugation, and the resulting protein suspension was sonicated on ice for 2 min at 40% amplitude and 2 s pulses.

#### Extract of Secreted Proteins (Secretome)

Extraction of *S. boydii* secretome was carried out following the methodology described in Buldain et al., 2019, with slight modifications. Briefly, 10^6^ conidia/ml were inoculated into PDB and grown for 24 h at 37ºC and 120 rpm. Fungal material was collected, washed twice with sterile PBS and cultured for 20 h at 37ºC and 120 rpm in PBS supplemented with 2% glucose in a proportion 1 g fungus: 1 mL medium. The culture was centrifuged, the supernatant filtered through a sterile gauze and then through a 0.22 µm membrane. Finally, the cell-free supernatant was concentrated using a 100,000 MWCO VIVASPIN centrifugal concentrator system (Sartorius, Göttingen, Germany). The resulting suspension was sonicated under the same conditions as the Total WCP extract described above.

#### Cell Surface Associated Proteins (Total CSP and Conidial CSP)

Proteins associated with the cell surface were collected by employing the protocol described by Pitarch et al. (2002), but with some modifications. To be precise, the culture of 5 x 10^5^ conidia/ml PDB for 24 h at 37ºC and 120 rpm was recovered by filtration and washed twice with PBS. Fungal material was resuspended in a volume of SDS extraction buffer and boiled for 10 min at 100ºC, cooled 5 min on ice, centrifuged and the supernatant containing Total CSP was recovered.

To obtain the Conidial CSP extract, the same procedure was carried out but using 10^8^ conidiospores as fungal material, which were directly resuspended in 1 ml of extraction buffer.

### Enzyme Linked Immunosorbent Assay (ELISA) for the Detection of *Scedosporium/Lomentospora*

Specific anti-*S. boydii* IgG was measured using a customized enzyme-linked immunosorbent assay (ELISA) method. ELISA was performed by coating wells of high binding micro test plates (Sarstedt, Nümbrecht, Germany) overnight at 4ºC with 10 µg/ml of protein extract diluted in sterilized PBS (100 µl per well). The following day the wells were washed three times with 200 µl PBS and blocked for 1 h at 37ºC by adding 200 µl of 5% (w/v) skimmed milk powder solution in PBS containing 0.05% (v/v) Tween 20 (PBST). Thereafter, three washes with PBST preceded the incubation at 37ºC for 1 h with 100 µl of human sera diluted 1:200 in PBST. In parallel, PBST without serum was added to some wells as negative control. Then these plates were washed three times with PBST, and 100 µl of HRP labeled anti-human-IgG (Sigma-Aldrich, St. Louis, MO, USA) diluted 1:10,000 in PBST was added to each well. After 1 h incubation at 37ºC, three washes with PBS preceded the incubation with 50 µl TMB substrate solution (Thermo Fisher, Waltham, MA, USA) for 30 min in the dark at 24ºC. Finally, the reaction was stopped by adding 50 µl of 0.5 M H_2_SO_4_ and absorbance (Abs) was measured at 450 nm using a Synergy TM HT plate reader (BioTek, Winooski, VT, USA).

A pool made up of the five Scedo+ sera selected was included in each experiment as a positive control to monitor batch-to-batch variations and to normalize the data after the study.

### Data Processing, Statistical Treatment, and Analysis

For data analysis, absorbance (Abs) values obtained in negative control wells were subtracted from remaining wells. Serum samples were measured in duplicate, and three replicates were performed for each ELISA experiment. With the aim of avoiding plate to plate bias variation, each Abs value was divided by the Abs value of the positive control included in all the plates and the result was expressed as Relative Abs.

Successive statistical analyses were run in SPSS Statistics software version 24 (IBM, Armonk, NY, USA) and the data plotted using Prism7 software (GraphPad, San Diego, CA, USA). Data distribution and its normality was studied by box plot analysis (see **Supplementary Material 1**). Two outlying values, classified into the Scedo- group, were identified and excluded from the analysis hereinafter. Normal distribution of data was detected by the Shapiro-Wilk test (< 50 samples) or the Kolmogorov-Smirnov test (> 50 samples), and homogeneity of the variance was proven by Levene test. Mean IgG response was compared between the three sera groups (Scedo+, Asp+, and Scedo-/Asp-) by performing a one-way analysis of variance (ANOVA) for normal distributed data or by Kruskal-Wallis for data with non-normal distribution, followed by Bonferroni’s multiple comparison test. Likewise, Scedo+ group’s mean specific IgG response was compared to the mean response of *Scedosporium* negative samples (Asp+ and Scedo-/Asp-) by performing Student’s *t* test or the Mann-Whitney U test for data with a normal and non-normal distribution, respectively. All the analyses were performed taking into account a confidence interval (CI) of 95%, for this a *p*-value < 0.05 was considered statistically significant.

The optimal diagnostic cut-off value to discriminate positive and negative results was selected by taking into consideration the following seven criteria defined in the bibliography (Habibzadeh et al., 2016; Mina et al., 2017; Unal, 2017): Youden Index (*J*), Concordance Probability Method (CZ), Index of Union (IU), Closest to [0.1] criteria (ER), Control mean Abs plus 2 standard deviation (SD) $\left(\bar{X}+2SD\right)$, and Sensitivity = Specificity (SE = SP), and the maximum positive likelihood ratio (MLR+).

To calculate the cut-off value of Abs by some of these criteria an analysis of the Receiver Operating Characteristic (ROC) curve was performed, and the performance of the test was checked based on the area under the ROC curve (AUC). To do this, Abs obtained with each serum sample of Scedo+ group were considered as Patient Values, meanwhile Abs of sera from Asp+ and Scedo-/Asp- groups were included in Control Values. In this way, the ROC area under the curve (AUC) was drawn with 95% CI.

Finally, the accuracy and test performance of the ELISA was evaluated by comparison with the “Gold Standard” method (mycological culture of the sputa), calculating validation parameters of Sensitivity (SE), Specificity (SP), Positive Predictive Value (PPV), Negative Predictive Value (NPV), and Efficiency (EFF). In addition, agreement between the two techniques was analyzed by Cohen’s Kappa index (K), which excludes the possibility of agreement occurring by chance.

## Results

### Fungal Prevalence in CF Patients’ Sputum Samples

**Figure 2:**
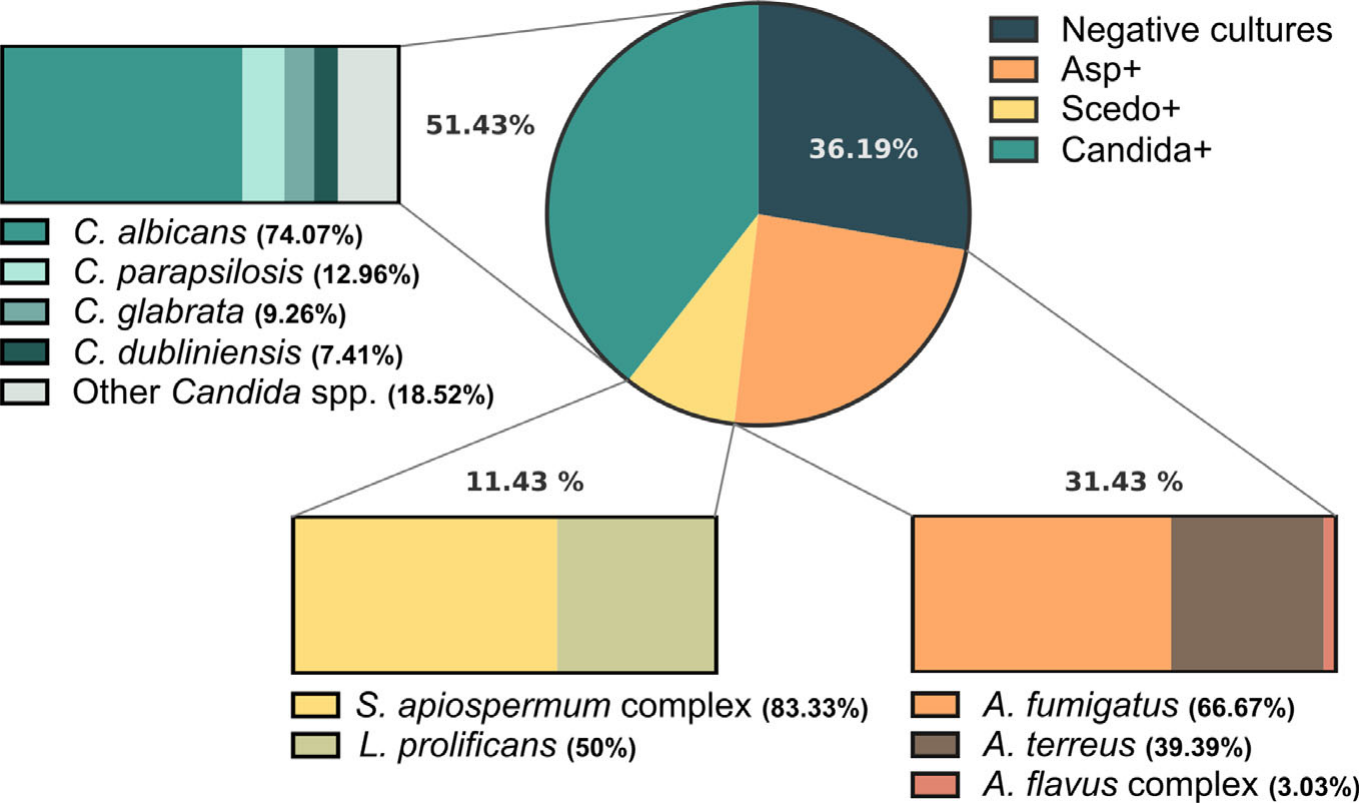
Frequency of isolation of fungal species from respiratory samples of Cystic Fibrosis (CF) patients when culturing sputum samples on Sabouraud gentamicin chloramphenicol agar and Modified Thayer Martin agar or Sabouraud chloramphenicol cycloheximide agar for the selective recovery of *Scedosporium* species from these polymicrobial samples. The circle depicts the general prevalence of *Aspergillus* spp., *Scedosporium*/*Lomentospora* and *Candida* spp., as well as the frequency of patients with negative cultures for these fungi: 31.43%, 11.43%, 51.43%, and 36.19%, respectively. Moreover, the species-specific prevalence is represented within the boxes.

The epidemiological study of the group of sera used in this work, carried out according to the microbiological examination of patients’ sputum (**Figure 2**), showed that *Candida* yeasts were detected in 51.43% of the patients, *C. albicans* being the most frequently isolated (74.07%). Regarding filamentous fungi, 31.43% of the patients resulted in positive cultures for species in the genus *Aspergillus, A. fumigatus* being the most prevalent (66.67%), followed by *Aspergillus terreus* (39.39%) and species of *Aspergillus flavus* complex (3.03%). Meanwhile, 11.43% of CF patients were positive for *Scedosporium/Lomentospora*, with the highest impact from the *S. apiospermum* species complex (83.33%) and a significant prevalence of *L. prolificans* (50%). Likewise, 36.19% of the population studied showed negative cultures for these three fungal pathogens.

### Reactivity of Human Sera Against *S. boydii* Protein Extracts

**Figure 3:**
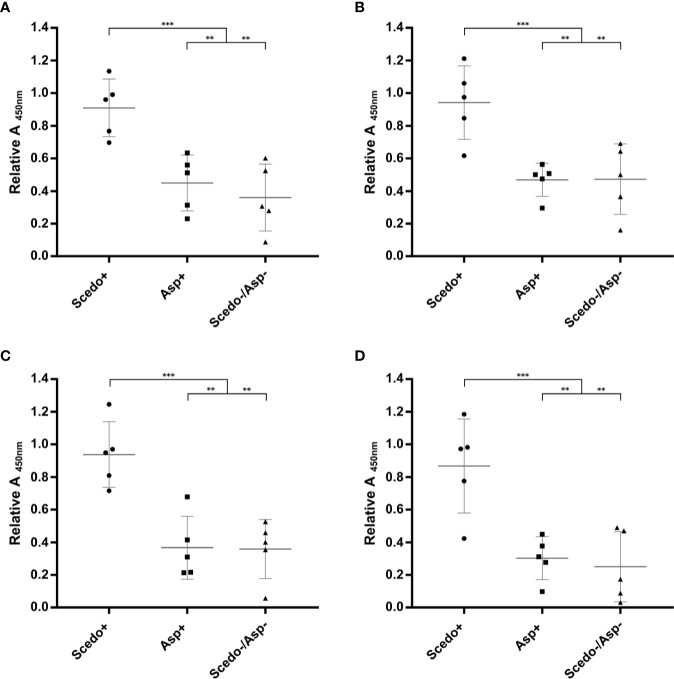
Screening of the immunoreactivity of Cystic Fibrosis (CF) patients’ sera against *Scedosporium boydii* protein extracts. Total WCP **(A)**, Secretome **(B)**, Total CSP **(C)**, and Conidial CSP **(D)**. Specific IgG antibody response is represented for the five sera selected from each group (Scedo+, Asp+ and Scedo-/Asp-) and against each protein extract, by relative Abs value at 450 nm obtained for each serum. Wide horizontal lines depict the media, and short horizontal lines represent the standard deviation (SD). Median value obtained for group Scedo+ is significantly different from that obtained for each of the other two groups, and for the sum of both (as negative samples) (^***^p < 0.001; ^**^p < 0.01).

With the aim of analysing the immunoreactive capacity and the discriminatory power of *S. boydii* protein extracts, specific IgG reactivity of five sera from different patients selected from each of the three groups of CF sera were measured by ELISA using four different types of protein extracts as antigen: Total WCP, Secretome, Total CSP, and Conidial CSP (**Figure 3**). In this sense, all extracts allowed the differentiation of the three groups, with the differences between Scedo+ and the other two groups, separately or together, statistically significant. From the four extracts, the best results were obtained with the Total WCP (**Figure 3A**) as discrimination of sera was the most accurate, and there was no overlap between groups.

**Figure 4:**
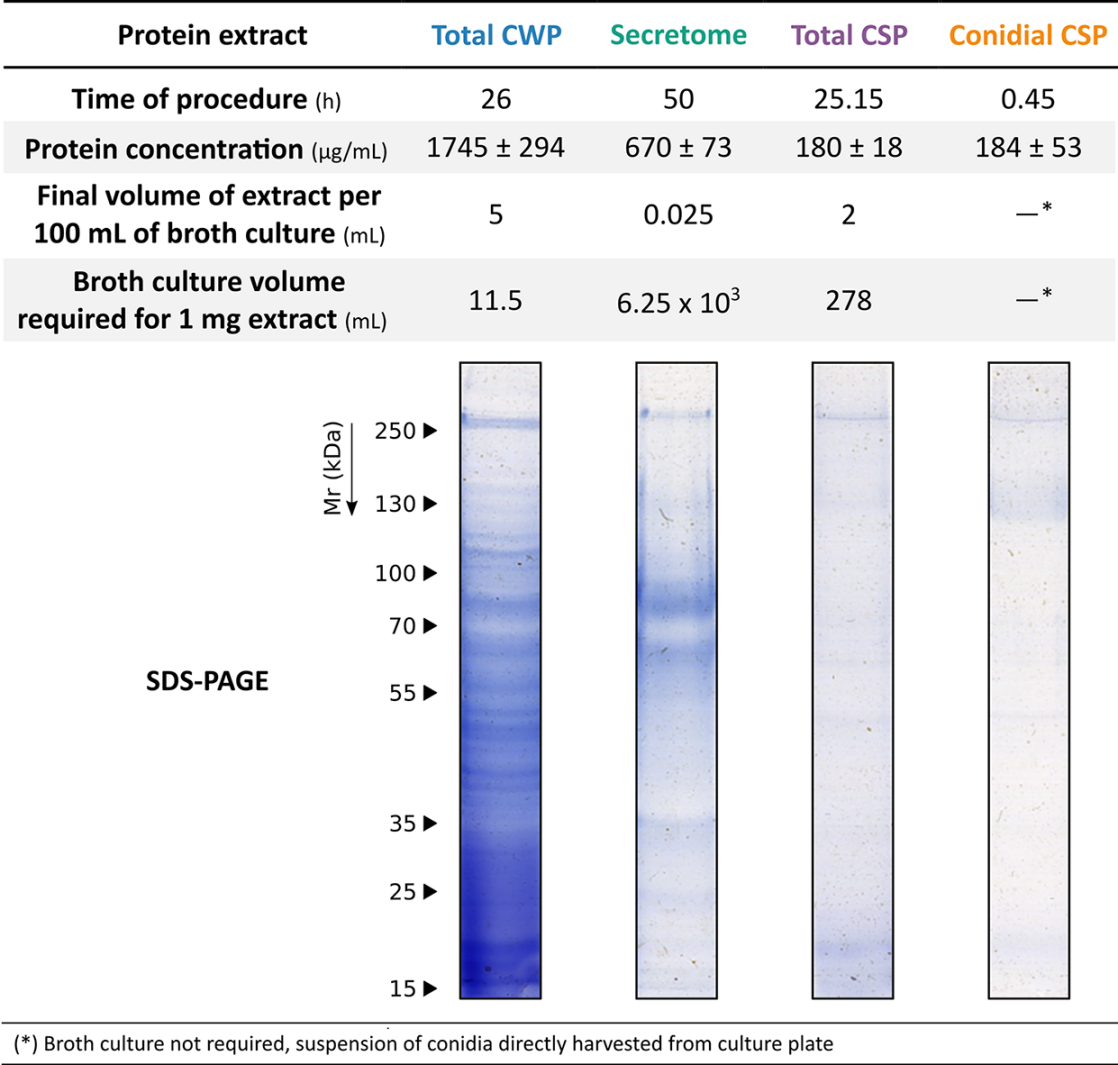
Evaluation of the efficiency of the methods for obtaining the different protein extracts of *S. boydii*. Approximate performance values of extraction methods: time of procedure, final volume of useful extract, protein concentration, and quality of the extract.

Although the secretome extract offered almost as accurate results, when evaluating the efficiency of the extraction methods (**Figure 4**) in terms of processing time, initial culture volume required, protein concentration, and volume of useful extract, the secretome extraction method was very time-consuming and yielded a lower concentration of protein.

### IgGs Cross-Reactivity Study With Total WCP Extract of *L. prolificans, A. fumigatus*, and *C. albicans*

**Figure 5:**
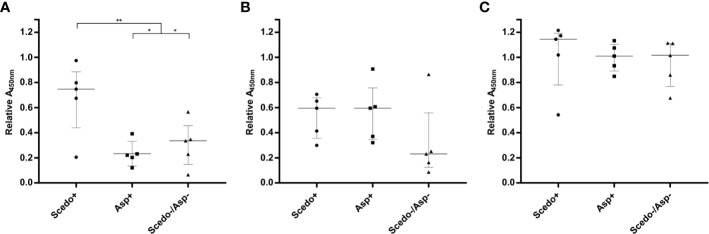
Immunoreactivity of CF patients’ serum IgGs to other fungal pathogens. Immunoreactivity of specific serum IgG against Total CWP of *L. prolificans* **(A)**, *A. fumigatus* **(B)**, and *C. albicans* **(C)** is represented as the relative Abs value at 450 nm obtained for each serum. Wide horizontal lines depict the median, and short horizontal lines represent the interquartile range (IQR), except for graph A where media (SD) is represented. Comparison of the median value among Scedo+ group and each of the other two groups, and for the sum of both as negative samples (^**^p < 0.01; ^*^p <0.05).

Selected sera were also tested against total WCP extract of the related species *L. prolificans*, and the most representative species of the two genera most frequently isolated in CF, *A. fumigatus*, and *C. albicans* (**Figure 5**). ELISA values showed that there is a high cross-reactivity between groups of patients when the antigens of *A. fumigatus* and *C. albicans* are used. Meanwhile, *L. prolificans* extract was able to discriminate between groups, with the differences being statistically significant. This demonstrates that there is high cross-reactivity between *Scedosporium* and *Lomentospora* because protein extracts of both fungi succeeded in discriminating Scedo+ patients (**Figure 5A**). Moreover, sera from Scedo+ groups seemed to cross react with *Aspergillus* extract (**Figure 5B**), although the contrary was not observed, as sera from Asp + did not detect Scedo+ extract at the same level (Fig 5A). In the case of the *C. albicans* extract, sera from the three groups showed high reactivity against it (**Figure 5C**).

### Total WCP Extract of *S. Boydii* as a Valuable Tool for Serological Detection of *Scedosporium*/*Lomentospora*

**Figure 6:**
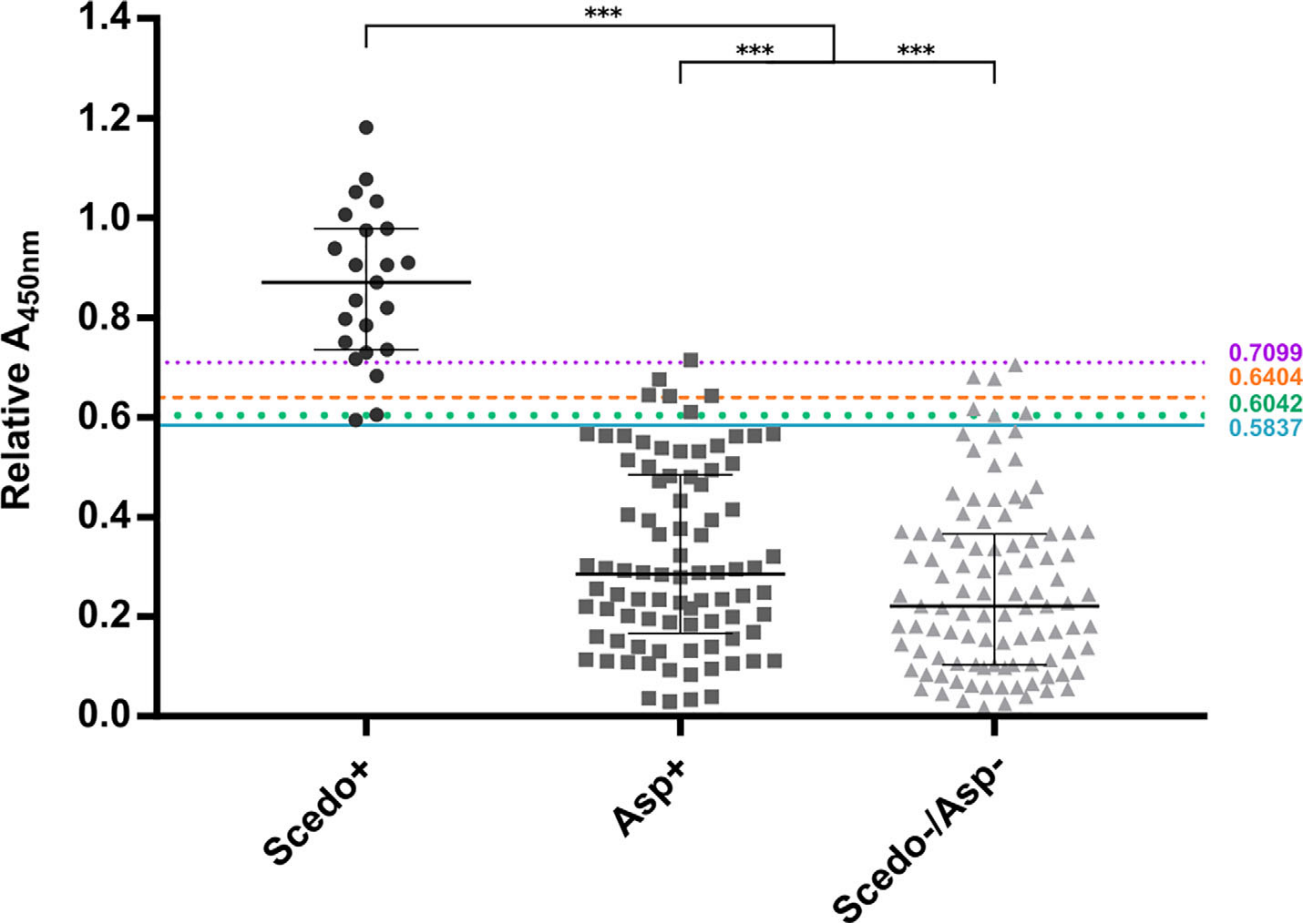
IgG response against *Scedosporium boydii* Total WCP extract of cystic fibrosis patients. Patients with positive sputum cultures for *Scedosporium/Lomentospora* are classified into group Scedo+, with positive cultures for *Aspergillus* spp. into group Asp+, and cultures without any recovery of these fungi into group Scedo-/Asp-. Antibody responses are represented by the relative Abs value at 450 nm obtained for each serum. The four cut-off values obtained are depicted as solid, dotted and dashed lines. Wide horizontal lines depict the median, and short horizontal lines represent the interquartile range (IQR). Median value obtained for group Scedo+ is significantly different from that obtained for each of the other two groups, and for the sum of both (as negative samples) (^***^p < 0.001).

In order to evaluate *S. boydii* Total WCP extract as a serodiagnostic tool for *Scedosporium*/*Lomentospora* detection, sera from the collection were tested individually using ELISA (**Figure 6**).

When plotting all the values obtained in the ELISA assay, the three categories of sera were well distinguished. Indeed, differences between the median value of the specific IgG response for group Scedo+ compared to that obtained for Asp+ and Scedo-/Asp- were statistically significant, and also differed significantly from that obtained considering all *Scedosporium* negative samples together (Asp+ and Scedo-/Asp-) (^***^p < 0.001).

**Figure 7:**
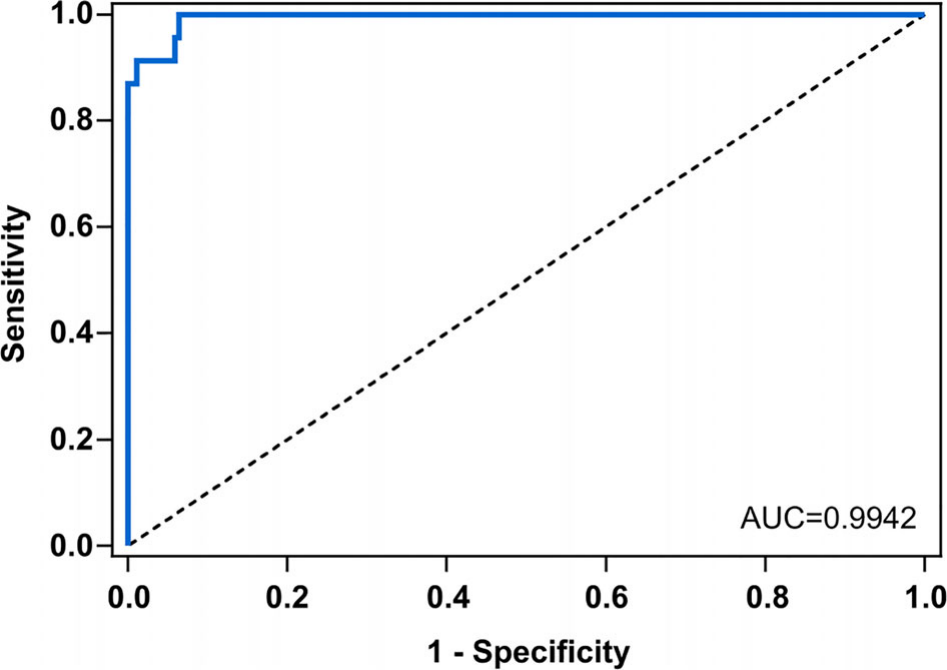
Receiving operating characteristics curve (ROC) for anti – *Scedosporium boydii* Total WCP IgG ELISA. Scedo+ patients vs. negative patients (Asp+ and Scedo-/Asp-). The area under the curve (AUC) for *S. boydii-*specific IgG was 0.9924. Line of identity is indicated with the dashed line.

The diagnostic performance of the test was assessed by conducting an ROC analysis that showed an AUC value of 0.9942, meaning a high discrimination capacity (**Figure 7**). Nevertheless, to assert whether a result was positive (detection of *Scedosporium*/*Lomentospora*) or negative, a decision threshold had to be established, so seven different criteria were used: *J*, CZ, IU, ER, $\bar{X}+2SD$, SE = SP, MLR+ (**Table 1**).

**Table 1:** Performance of *Scedosporium boydii* specific IgG at different cut-off values with 25 serum samples of Cystic Fibrosis patients with *Scedosporium*/*Lomentospora* positive sputum cultures and 190 negative sera.

Cut-off value (Relative A_450nm_)	Criteria	Test validation parameters (%)					
		SE	SP	EFF	PPV*	NPV*	K
0.5837	*J*, CZ, IU, ER	100	93.58	94.29	65.71	100	0.76
0.6042	SE=SP	95.65	94.12	94.29	66.67	99.44	0.75
0.6404	$\left(\bar{X}+2SD\right)$	91.30	95.72	95.24	72.41	98.90	0.78
0.7099	MLR+	86.96	99.47	98.10	95.24	98.41	0.89

J, Youden Index; CZ, Concordance Probability Method; IU, Index of Union; ER, Closest to [0,1]; $\bar{X}$, mean value of control sera absorbance; SD, standard deviation; MLR+, maximum positive likelihood ratio; SE, sensitivity; SP, specificity; EFF, efficiency or correct classification rate; PPV, predictive value of positive test; NPV, predictive value of negative test; K, Cohen’s Kappa index.

(*) Theoretical values considering a prevalence of Scedosporium/Lomentospora of 11.43% observed in this study.

According to the above-mentioned criteria, four cut-off values were identified: Abs_450nm_ > 0.5837 using *J*, CZ, IU, and ER criteria, and Abs_450nm_ > 0.6042, > 0.6404 and > 0.7099 using SE = SP, $\bar{X}+2SD$, and MLR+, respectively. When evaluating the test performance, the SE was ≥ 86.9%, SP ≥ 93.6% and the PVN ≥ 98.4% regardless of the cut-off point selected. However, the PPV suffers a large variation, from 65%–95%, depending on the cut-off value. The K index was calculated as 0.76, 0.75, and 0.78 for the 0.5837, 0.6042, and 0.6404 cut-offs respectively, indicating a substantial agreement excluding chance. For the 0.7099 cut-off the K index calculated was 0.89 which indicates an almost perfect agreement. The cut-offs and the corresponding validation parameters are detailed in **Table 1**.

### Monitoring of Specific IgG Levels Against *Scedosporium*

**Figure 8:**
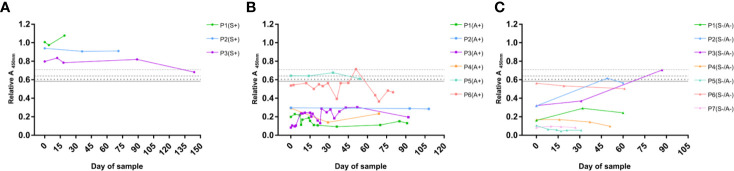
Monitoring of specific IgG serological response of CF patients against *S. boydii*. Example of representative patients of the Scedo+ group **(A)**, Asp+ group **(B)**, and Scedo-/Asp- group **(C)**. Relative Abs values of the ELISA test using a total WCP of *S. boydii* are depicted on a time scale.

In order to determine the utility of the designed serological test, not only for serodiagnosis using a single sample point but also in the monitoring of CF patients, the Abs value of several serum samples corresponding to the same patient were plotted in a time-dependent manner according to the date of sample. The monitoring of patients (P) with at least three serum samples obtained over a period of more than 15 days is shown in **Figure 8** and distributed in three different graphs according to the CF group in which they were previously classified. In this way, the evolution of the humoral IgG specific response against *S. boydii* can be observed and therefore, the evolution of the disease. In fact, some interesting trends where found when looking at the patients represented.

Scedo+ monitored patients (**Figure 8A**) showed a high specific IgG response, which in all cases exceeded the cut-off values determined in this study. Specifically, P1 and P2 showed values that remained stable as clearly positive over the sampling interval. Conversely, P3 experienced an initial period of stability, but the final sample value decreased and crossed the threshold towards a negative outcome, and therefore, suggests a possible clinical improvement that should be confirmed with subsequent additional samples.

Regarding the Asp+ group (**Figure 8B**) most of the serological profiles plotted corresponded to representative patients from the group, whose values were clearly under the diagnostic threshold. Nevertheless, two of the monitored patients showed a fairly high humoral response. P6 was close to the cut-off, even in certain sample times that crossed the threshold, while P5 exhibited a serological response that matches the profile of culture-confirmed Scedo+ patients. In this sense, these patients should remain under surveillance since they are at risk of presenting an undiagnosed *Scedosporium* colonization.

Finally, the monitoring of the patients from the Scedo-/Asp- group is illustrated in **Figure 8C**. Although most of the patients maintained negative results during the sampling period, P1, P2, and P3 showed intriguing serological profiles due to the high values detected. While P6 started with values close to the threshold but eventually developed a downward trend, P2 and P3 started the sampling with clearly negative results, however, high positive values were obtained in later samples. Which in turn, indicates that these patients, and especially P3, need to be carefully monitored since they may have an undetected presence of *Scedosporium*.

## Discussion

The range of fungal species detected in CF patients continues to increase in line with new discoveries in diagnostic methodologies (Tracy & Moss, 2018). The development of selective culture media has become a cornerstone for improving the classic gold standard of culture-based diagnosis, enhancing the recovery of low prevalence and/or slow growing fungal pathogens (Pham et al., 2015; Hong et al., 2017; Coron et al., 2018). However, there are still huge limitations that hinder the correct detection of these fungi, such as the lack of standardized guidelines for processing respiratory samples and the absence of commercial available culture media designed for these challenging isolations (Chen et al., 2017). On the other hand, serological tests have led to non-culture-based diagnosis of fungal infections since the 1950s because of their advantages, such as the ease of minimally invasive sample collection (Richardson and Page, 2018). Unfortunately, at present, there is a lack of specific serological kits for low-prevalence fungal pathogens that enable rapid and easy detection.

For many years, efforts have been focused on *Aspergillus fumigatus* as it is the most prevalent filamentous fungi in CF airways (Richardson & Page, 2017). In this sense, crude antigenic extracts from conidia and hyphae have been widely used for *Aspergillus* serological detection (Page et al., 2016). On the other hand, species from *Scedosporium* genus are gaining more and more attention because of their high chronicity in CF airways as well as their associated pathogenicity. However, despite their disturbing clinical relevance, accurate detection methods that allow an adequate diagnosis of these threatening pathogens is currently lacking (Chen et al., 2017). Mycological culturing continues to be the most widespread method. This requires the use of selective media that are not available everywhere, and incubation times for these slow-growers are excessively long (Coron et al., 2018). This problem is reflected in the epidemiological studies that suffer worrying variations in the prevalence rates, caused to some extent, by the use of different and non-standardized procedures. In fact, there is a growing awareness of this issue and this has led to the publication of research into the frequency variation depending on the detection method employed (Borman et al., 2010; Sedlacek et al., 2015; Hong et al., 2017; Boyle et al., 2018; Hedayati et al., 2019). These diagnostic hurdles in turn result in delayed diagnosis, and consequently, a late introduction of an effective treatment. Therefore, considering the mounting concern about the critical need for standardized and reliable detection methods, efforts must be made to develop rapid and robust tests to ensure an early detection of *Scedosporium*, and consequently the establishment of an effective treatment and patient monitoring. With this in mind, the aim of this study was to analyse the utility of different protein extracts of *S. boydii* for a serological detection of *Scedosporium*/*Lomentospora* in CF patients by ELISA.

To achieve this, 212 serum samples corresponding to 105 CF patients were used. Patients were classified into three groups, Scedo+, Asp+, and Scedo-/Asp-, based on the fungus isolated from the sputum, *Scedosporium, Aspergillus*, or neither, respectively. Mycological cultures showed that *Candida* was the most frequently isolated fungus among yeasts, and *Aspergillus* among filamentous fungi. Nevertheless, *Scedosporium* species were isolated from 11.43% of CF patients’ sputa, these results were in agreement with the prevalence rates of 8%–16% published in different epidemiological studies (Cimon et al., 2000; Blyth et al., 2010; Schwarz et al., 2017). Moreover, species of *S. apiospermum* complex were isolated in 85.33% of Scedo+ patients, which is consistent with the *Scedosporium* species distribution in CF, since *S. apiospermum* and *S.boydii* are the most frequently isolated species (Bouchara et al., 2019). Finally, *L. prolificans* was detected in 50% of Scedo+ patients, which is a notoriously high prevalence rate when compared with the worldwide incidence data (0%–40%), but it is in concert with the geographical restriction of the fungus, Spain and Australia being the countries with the highest incidence (Seidel et al., 2019).

The first step in the design of the ELISA for the serological detection of *Scesdosporium/Lomentospora* was the selection of an easy-to-obtain protein extract with a good level of discrimination. Some research has deciphered potential *Scedosporium* virulence markers (reviewed in Santos et al., 2009) with different cellular locations that are related to both morphological phases (hypha and conidium). Bearing in mind the wide variety of antigens, the immunoreactivity and discriminatory capacity of four *S. boydii* protein extracts, which included total protein extract (Total WCP), secretome extract, cell surface associated proteins (Total CSP), and cell surface associated proteins only from conidia (Conidia CSP), were compared by indirect ELISA against fifteen patients (five from each CF group). The results obtained with the four extracts showed that they were able to discriminate Scedo+ sera from Asp+ and Scedo-/Asp- groups. However, of the four extracts, Total WCP was selected to continue with the study as it was the one with the highest output in terms of protein concentration per mL of fungal culture used, and the discriminatory capacity was the best because none of the five patients tested in the Scedo+ group overlapped with any of the other two groups.

Among the discarded extracts, secretome was expected to be interesting because of the chronicity of *Scedosporium* colonizations of CF airways and the specificity of the secreted proteins according to bibliography (Santos et al., 2009; Bertrand et al., 2010). Moreover, metalloproteases such as superoxide dismutase, some proteolytic enzymes, as well as ectophosphatases are secreted to the external environment and play a protective role for the fungus and orchestrate the cleavage of key host components (Larcher et al., 1996; Da Silva et al., 2006; Da Silva et al., 2012). In agreement with this idea, the results obtained using this extract were very good, but similar to the ones obtained with the Total WCP and, additionally, there were some problems with gathering high protein concentrations, a long and complex process was necessary to obtain an extract with low yields.

Regarding Total CSP and Conidial CSP, it is well known that some cell wall associated proteins of *Scedosporium*, such as glucans, peptidorhamnomanns and glucosylceramides, show a high immunoreactive capacity (Pinto et al., 2004; Da Silva Xisto et al., 2019). Moreover, since the typical entry of the fungus into the host body is through inhalation of conidia, it is reasonable for proteins of the cell wall of the conidium to play an important role in the host-pathogen interactions and colonization/infection of airways (Buldain et al., 2016). Consequently, as with the secretome, the results obtained were also good but yielded low protein concentration and in addition, some overlapping between groups with the Conidial CSP was observed. This cross-reactivity could be explained by the fact that some surface proteins of the conidia are common to different moulds, are important for the survival of the fungus, and possess immune-modulatory functions (Voltersen et al., 2018).

Hence, further experiments were carried out using Total WCP. Crude antigenic extracts have been widely used in immunodiagnostic systems, some of them being currently commercially available for the detection of *Aspergillus* (Page et al., 2016; Richardson and Page, 2017). The advantage of these kinds of extract is that they involve every interesting antigen. On the contrary, one of their main handicaps is their cross-reactivity against panfungal antigens of closely related fungi (Buldain et al., 2019). In this sense, biochemical studies try to characterize specific proteins for diagnostic purposes, but as little is known about the physiology and biochemistry of *Scedosporium*, and the number of studies performed in the field is small, only a few proteins of interest have been characterized. Among these are a serine protease from the subtilisin family, two enzymes involved in ROX detoxification, cytosolic Cu,Zn-superoxide dismutase, a catalase and some heat shock proteins (Santos et al., 2009).

Moreover, the potential of crude antigenic extracts of *Scedosporium/Lomentospora*, which is the first step in the serodiagnosis race, has not yet been evaluated. In this way, Total WCP was tested against the totality of sera by the same optimized ELISA assay, and the results show that it was able to discriminate the Scedo+ patients successfully. In this sense, ROC AUC analysis possesses a good discriminatory capacity for the test (AUC=0.9942) but assessing the criteria for cut-off determination (Habibzadeh et al., 2016; Unal, 2017), four threshold values were represented. The most affected parameter was the theoretical PPV*, because a PPV* of 65% was obtained with the lowest threshold values but it increased to 95% in the highest one. Theoretical NPV*, efficiency and specificity values remained more or less stable in all the cases, while sensitivity varied from 86%–100% with the highest and the lowest cut-offs, respectively. Despite the variations observed, the ELISA assay showed a remarkably high specificity, which means an absolute capacity to discriminate CF patients with no presence of *Scedosporium*. Moreover, it was observed that being stricter with the selection of the cut-off value resulted in improved ability to detect *Scedosporium* positive patients. Nevertheless, choosing one cut-off value may be risky considering the limited number of Scedo+ samples. In this sense, increasing this sample population might be helpful to establish a definitive cut-off point.

Turning to the commercial kits for *Aspergillus* detection that show a 75%–96% sensitivity (Richardson and Page, 2017), the developed test exhibits a remarkable clinical usefulness considering that values of 86 - 100% of sensitivity and 93%–99% of specificity were obtained. Moreover, it is worth mentioning that *Lomentospora*- positive culture patients also showed a specific response against *S. boydii* extract, so the developed ELISA platform is a valuable tool to detect the intrinsic multi-resistant fungus *L. prolificans* as well. In the last few years, other authors have made efforts to shed light on serodiagnosis of *Scedosporium*. In fact, Bouchara and co-workers in 2017 developed an ELISA with two recombinant proteins, *Scedosporium* catalase A1 and cytosolic Cu,Zn-superoxyde dismutase, described as antigens with diagnostic utility in *Aspergillus*. In this study, they managed to detect *Scedosporium* infections, and differentiate it from an *Aspergillus* infection. Moreover, precipitin assays can be performed but they take up to one week for the results to be obtained, lack sensitivity (Fujiuchi et al., 2016), and can only be performed in a few specialized laboratories (Cimon et al., 2000).

Finally, the study of the ELISA test as a patients’ monitoring tool showed the value of the technique for the observation of the evolution of the fungal presence since the humoral response of the patient can be tracked over time, although this point should be studied in more depth in the future. Nevertheless, the best diagnosis is the variety of tests with different purposes that complement each other and offer a real vision of the patient’s condition and evolution. In this sense, our research group aimed to explore new diagnostic resources by developing an indirect ELISA test using *S. boydii* whole cell protein extract. Detecting humoral response against *Scedosporium* in CF patients regardless the result of the culture may be helpful for clinicians to maintain these patients under surveillance, and to anticipate the establishment of their antifungal treatment.

## Conclusions

In this study a crude antigenic extract of *S. boydii* was selected to detect *Scedosporium/Lomentospora* serologically. The ELISA test developed is able to detect the *Scedosporium* spp. and *L. prolificans* in CF patients’ sera, with a very high sensitivity and specificity, up to 100% and 99%, respectively. Thus, this ELISA platform offers a rapid, low-cost and easy solution to detect these elusive fungi through minimally invasive sampling, with high output and specificity, and allows the monitoring of the evolution of the infection, the recovery and the effectiveness of the antifungal therapy. In spite of the potential of these results, more research is needed in this field to detect specific antigens from the fungal extract to improve sensitivity and specificity, to minimize cross-reactivity with other closely related fungal pathogens, and ultimately to reach a standardized method. Nevertheless, it is worth bearing in mind that the best diagnosis is achieved with a combination of methods that allow a complete vision of the infection.

## Data Availability Statement

The raw data supporting the conclusions of this article will be made available by the authors, without undue reservation.

## Ethics Statement

The studies involving human participants were reviewed and approved by Ethics Committee from the UPV/EHU. The patients/participants provided their written informed consent to participate in this study.

## Author Contributions

LM-S, IB, LA-F, and MA carried out the experiments. J-PB and MM-G obtained and classified the sera samples. AR, FH and AR-G conceived the experiments and supervised the work. LM-S, AR, FH, and AR-G analyzed the data. LM-S and AR-G wrote the manuscript. All authors contributed to the article and approved the submitted version.

## Funding

This research was funded by the Basque Government, grant number IT1362-19. IB, LM-S, and LA-F received a predoctoral fellowship from the Basque Government. The funders had no role in the design of the study; in the collection, analyses, or interpretation of data; in the writing of the manuscript, or in the decision to publish the results.

## Conflict of Interest

The authors declare that the research was conducted in the absence of any commercial or financial relationships that could be construed as a potential conflict of interest.

## References

[B1] Bertrand S. Bouchara J. P. Venier M. C. Richomme P. Duval O. Larcher G. (2010). N(α)-methyl coprogen B, a potential marker of the airway colonization by *Scedosporium apiospermum* in patients with cystic fibrosis. Med. Mycol. 48, S98–S107. 10.3109/13693786.2010.503972 21067336

[B2] Blyth C. C. Harun A. Middleton P. G. Sleiman S. Lee O. Sorrell T. C. (2010). Detection of occult *Scedosporium* species in respiratory tract specimens from patients with cystic fibrosis by use of selective media. J. Clin. Microbiol. 48, 314–316. 10.1128/JCM.01470-09 19906904 PMC2812274

[B3] Borman A. M. Palmer M. D. Delhaes L. Carrère J. Favennec L. Ranque S. (2010). Lack of standardization in the procedures for mycological examination of sputum samples from CF patients: A possible cause for variations in the prevalence of filamentous fungi. Med. Mycol. 48, S88–S97. 10.3109/13693786.2010.511287 21067335

[B4] Bouchara J. P. Le Govic Y. Kabbara S. Cimon B. Zouhair R. Hamze M. (2019). Advances in understanding and managing *Scedosporium* respiratory infections in patients with cystic fibrosis. Expert Rev. Respir. Med. 14, 259–273. 10.1080/17476348.2020.1705787 31868041

[B5] Boyle M. Moore J. E. Whitehouse J. L. Bilton D. Downey D. G. (2018). Laboratory diagnosis and characterization of fungal disease in patients with cystic fibrosis (CF): A survey of current UK practice in a cohort of clinical microbiology laboratories. Mycopathologia. 183, 723–729. 10.1007/s11046-018-0251-z 29500636

[B6] Buldain I. Ramirez-Garcia A. Pellon A. Antoran A. Sevilla M. J. Rementeria A. (2016). Cyclophilin and enolase are the most prevalent conidial antigens of *Lomentospora prolificans* recognized by healthy human salivary IgA and cross-react with *Aspergillus fumigatus*. Proteomics Clin. Appl. 10, 1058–1067. 10.1002/prca.201600080 27485921

[B7] Buldain I. Pellon A. Zaldibar B. Antoran A. Martin-Souto L. Aparicio-Fernandez L. (2019). Study of humoral responses against *Lomentospora*/S*cedosporium* spp. and *Aspergillus fumigatus* to identify *L. prolificans* antigens of interest for diagnosis and treatment. Vaccines 7, 212. 10.3390/vaccines7040212 PMC6963885 31835471

[B8] Castellani C. Assael B. M. (2017). Cystic fibrosis: a clinical view. Cell Mol. Life Sci. 74, 129–140. 10.1007/s00018-016-2393-9 27709245 PMC11107741

[B9] Chen S. C.-A. Meyer W. Pashley C. H. (2017). Challenges in laboratory detection of fungal pathogens in the airways of cystic fibrosis patients. Mycopathologia. 183, 89–100. 10.1007/s11046-017-0150-8 28589247

[B10] Cimon B. Carrère J. Vinatier J. F. Chazalette J. P. Chabasse D. Bouchara J. P. (2000). Clinical significance of *Scedosporium apiospermum* in patients with cystic fibrosis. Eur. J. Clin. Microbiol. Infect. Dis. 19, 53–56. 10.1007/s100960050011 10706182

[B11] Coron N. Pihet M. Fréalle E. Lemeille Y. Pinel C. Pelloux H. (2018). Toward the standardization of mycological examination of sputum samples in cystic fibrosis: Results from a french multicenter prospective study. Mycopathologia. 183, 101–117. 10.1007/s11046-017-0173-1 28748285

[B12] Da Silva B. A. Dos Santos A. L. S. Barreto-Bergter E. Pinto M. R. (2006). Extracellular peptidase in the fungal pathogen *Pseudallescheria boydii*. Curr. Microbiol. 53, 18–22. 10.1007/s00284-005-0156-1 16775782

[B13] Da Silva B. A. Sodré C. L. Souza-Gonçalves A. L. Aor A. C. Kneipp L. F. Fonseca B. B. (2012). Proteomic analysis of the secretions of *Pseudallescheria boydii*, a human fungal pathogen with unknown genome. J. Proteome Res. 11, 172–188. 10.1021/pr200875x 22142336

[B14] Da Silva Xisto M. II Henao J. E. M. Dos Santos Dias L. Santos G. M. P. De Oliveira Rocha Calixto R. Bernardino M. C. (2019). Glucosylceramides from *Lomentospora prolificans* induce a differential production of cytokines and increases the microbicidal activity of macrophages. Front. Microbiol. 10, 554. 10.3389/fmicb.2019.00554 30967849 PMC6440385

[B15] Dyballa N. Metzger S. (2009). Fast and sensitive colloidal Coomassie G-250 staining for proteins in polyacrylamide gels. J. Vis. Exp. 30, 2–5. 10.3791/1431 PMC3149902 19684561

[B16] Elborn J. S. (2016). Cystic fibrosis. Lancet 388, 2519–2531. 10.1016/S0140-6736(16)00576-6 27140670

[B17] Fujiuchi S. Fujita Y. Suzuki H. Doushita K. Kuroda H. Takahashi M. (2016). Evaluation of a quantitative serological assay for diagnosing chronic pulmonary aspergillosis. J. Clin. Microbiol. 54, 1496–1499. 10.1128/JCM.01475-15 27008878 PMC4879291

[B18] Habibzadeh F. Habibzadeh P. Yadollahie M. (2016). On determining the most appropriate test cut-off value: The case of tests with continuous results. Biochem. Med. 26, 297–307. 10.11613/BM.2016.034 PMC5082211 27812299

[B19] Hedayati M. T. Tavakoli M. Maleki M. Heidari S. Mortezaee V. Gheisari M. (2019). Fungal epidemiology in cystic fibrosis patients with a special focus on *Scedosporium* species complex. Microb. Pathog. 129, 168–175. 10.1016/j.micpath.2019.02.009 30742949

[B20] Hong G. Miller H. B. Allgood S. Lee R. Lechtzin N. Zhang S. X. (2017). Use of selective fungal culture media increases rates of detection of fungi in the respiratory tract of cystic fibrosis patients. J. Clin. Microbiol. 55, 1122–1130. 10.1128/JCM.02182-16 28100601 PMC5377839

[B21] Larcher G. Cimon B. Symoens F. Tronchin G. Chabasse D. Bouchara J. P. (1996). A 33 kDa serine proteinase from *Scedosporium apiospermum*. Biochem. J. 315, 119–126. 10.1042/bj3150119 8670095 PMC1217159

[B22] LiPuma J. J. (2010). The changing microbial epidemiology in cystic fibrosis. Clin. Microbiol. Rev. 23, 299–323. 10.1128/CMR.00068-09 20375354 PMC2863368

[B23] Martín-Gómez M. T. (2020). Taking a look on fungi in cystic fibrosis: More questions than answers. Rev. Iberoam Micol. 37, 17–23. 10.1016/j.riam.2019.10.004 31928888

[B24] Mello T. P. Bittencourt V. C. B. Liporagi-Lopes L. C. Aor A. C. Branquinha M. H. Santos A. L. S. (2019). Insights into the social life and obscure side of *Scedosporium*/*Lomentospora* species: ubiquitous, emerging and multidrug-resistant opportunistic pathogens. Fungal Biol. Rev. 33, 16–46. 10.1016/j.fbr.2018.07.002

[B25] Mina S. Staerck C. Marot A. Godon C. Calenda A. Bouchara J.-P. P. (2017). *Scedosporium boydii* CatA1 and SODC recombinant proteins, new tools for serodiagnosis of *Scedosporium* infection of patients with cystic fibrosis. Diagn. Microbiol. Infect. Dis. 89, 282–287. 10.1016/j.diagmicrobio.2017.08.013 28974395

[B26] Mirtajani S. Farnia P. Hassanzad M. Ghanavi J. Farnia P. Velayati A. (2017). Geographical distribution of cystic fibrosis; The past 70 years of data analyzis. BioMed. Biotech. Res. J. 1, 105–112. 10.4103/bbrj.bbrj_81_17

[B27] Page I. D. Richardson M. D. Denning D. W. (2016). Comparison of six *Aspergillus*-specific IgG assays for the diagnosis of chronic pulmonary aspergillosis (CPA). J. Infect. 72, 240–249. 10.1016/j.jinf.2015.11.003 26680697

[B28] Pellon A. Ramirez-Garcia A. Buldain I. Antoran A. Rementeria A. Hernando F. L. (2016). Immunoproteomics-Based analysis of the immunocompetent serological response to *Lomentospora prolificans*. J. Proteome Res. 15, 595–607. 10.1021/acs.jproteome.5b00978 26732945

[B29] Pellon A. Ramirez-Garcia A. Buldain I. Antoran A. Martin-Souto L. Rementeria A. (2018). Pathobiology of *Lomentospora prolificans*: could this species serve as a model of primary antifungal resistance? Int. J. Antimicrob. Agents. 51, 10–15. 10.1016/j.ijantimicag.2017.06.009 28669833

[B30] Pham T. Giraud S. Schuliar G. Rougeron A. Bouchara J. P. (2015). Scedo-Select III: A new semi-selective culture medium for detection of the *Scedosporium apiospermum* species complex. Med. Mycol. 53, 512–519. 10.1093/mmy/myv015 25841055

[B31] Pinto M. R. De Sá A. C. M. Limongi C. L. Rozental S. Santos A. L. S. Barreto-Bergter E. (2004). Involvement of peptidorhamnomannan in the interaction of *Pseudallescheria boydii* and HEp2 cells. Microbes Infect. 6, 1259–1267. 10.1016/j.micinf.2004.07.006 15555531

[B32] Pitarch A. Sánchez M. Nombela C. Gil C. (2002). Sequential fractionation and two-dimensional gel analysis unravels the complexity of the dimorphic fungus Candida albicans cell wall proteome. Mol. Cell. Proteomics 12, 9677–982. 10.1074/mcp.m200062-mcp200 12543933

[B33] Ramirez-Garcia A. Pellon A. Rementeria A. Buldain I. Barreto-Bergter E. Rollin-Pinheiro R. (2018). *Scedosporium* and *Lomentospora*: an updated overview of underrated opportunists. Med. Mycol. 56, 102–125. 10.1093/mmy/myx113 29538735

[B34] Richardson M. D. Page I. D. (2017). *Aspergillus* serology: Have we arrived yet? Med. Mycol. 55, 48–55. 10.1093/mmy/myw116 27816904

[B35] Richardson M. Page I. (2018). Role of Serological Tests in the Diagnosis of Mold Infections. Curr. Fungal Infect. Rep. 12, 127–136. 10.1007/s12281-018-0321-1 30294405 PMC6153857

[B36] Rowe S. M. Miller S. Sorscher E. J. (2005). Cystic fibrosis. N. Engl. J. Med. 352, 1992–2001. 10.1056/NEJMra043184 15888700

[B37] Santos A. L. S. Bittencourt V. C. B. Pinto M. R. Silva B. A. Barreto-Bergter E. (2009). Biochemical characterization of potential virulence markers in the human fungal pathogen *Pseudallescheria boydii*. Med. Mycol. 47, 375–386. 10.1080/13693780802610305 19235547

[B38] Schwarz C. Brandt C. Antweiler E. Krannich A. Staab D. Schmitt-Grohé S. (2017). Prospective multicenter German study on pulmonary colonization with *Scedosporium*/*Lomentospora* species in cystic fibrosis: Epidemiology and new association factors. PloS One 12, 171485. 10.1371/journal.pone.0171485 PMC5298894 28178337

[B39] Schwarz C. Vandeputte P. Rougeron A. Giraud S. Dugé de Bernonville T. Duvaux L. (2018). Developing collaborative works for faster progress on fungal respiratory infections in cystic fibrosis. Med. Mycol. 56, 42–59. 10.1093/mmy/myx106 29538733

[B40] Sedlacek L. Graf B. Schwarz C. Albert F. Peter S. Würstl B. (2015). Prevalence of *Scedosporium* species and *Lomentospora prolificans* in patients with cystic fibrosis in a multicenter trial by use of a selective medium. J. Cyst Fibros. 14, 237–241. 10.1016/j.jcf.2014.12.014 25595044

[B41] Seidel D. Meißner A. Lackner M. Piepenbrock E. Salmanton-García J. Stecher M. (2019). Prognostic factors in 264 adults with invasive *Scedosporium* spp. and *Lomentospora prolificans* infection reported in the literature and FungiScope^®^. Crit. Rev. Microbiol. 45, 1–21. 10.1080/1040841X.2018.1514366 30628529

[B42] Soret P. Vandenborght L. Francis F. Coron N. Enaud R. Mucofong T. (2020). Respiratory mycobiome and suggestion of inter-kingdom network during acute pulmonary exacerbation in cystic fibrosis. Sci. Rep. 10, 3589. 10.1038/s41598-020-60015-4 32108159 PMC7046743

[B43] Symoens F. Knoop C. Schrooyen M. Denis O. Estenne M. Nolard N. (2006). Disseminated *Scedosporium apiospermum* infection in a cystic fibrosis patient after double-lung transplantation. J. Heart Lung Transplant. 25, 603–607. 10.1016/j.healun.2005.12.011 16678041

[B44] Tracy M. C. Moss R. B. (2018). The myriad challenges of respiratory fungal infection in cystic fibrosis. Pediatr. Pulmonol. 53, S75–S85. 10.1002/ppul.24126 29992775

[B45] Unal I. (2017). Defining an optimal cut-point value in ROC analysis: An alternative approach. Comput. Math Methods Med. 2017, 3762651. 10.1155/2017/3762651 28642804 PMC5470053

[B46] Voltersen V. Blango M. G. Herrmann S. Schmidt F. Heinekamp T. Strassburger M. (2018). Proteome analysis reveals the conidial surface protein CcpA essential for virulence of the pathogenic fungus *Aspergillus*. mBio 9, 1–18. 10.1128/mBio.01557-18 PMC6168859 30279286

