# Supplementary material for: ELISA Test for the Serological Detection of Scedosporium/Lomentospora in Cystic Fibrosis Patients
Source: Front Cell Infect Microbiol. 2020 Nov 26;10:602089. doi: 10.3389/fcimb.2020.602089 (PMC7726441; doi:10.3389/fcimb.2020.602089)
Supplement: Supplementary file 1 [file DataSheet_1.docx]

Supplementary Material

***
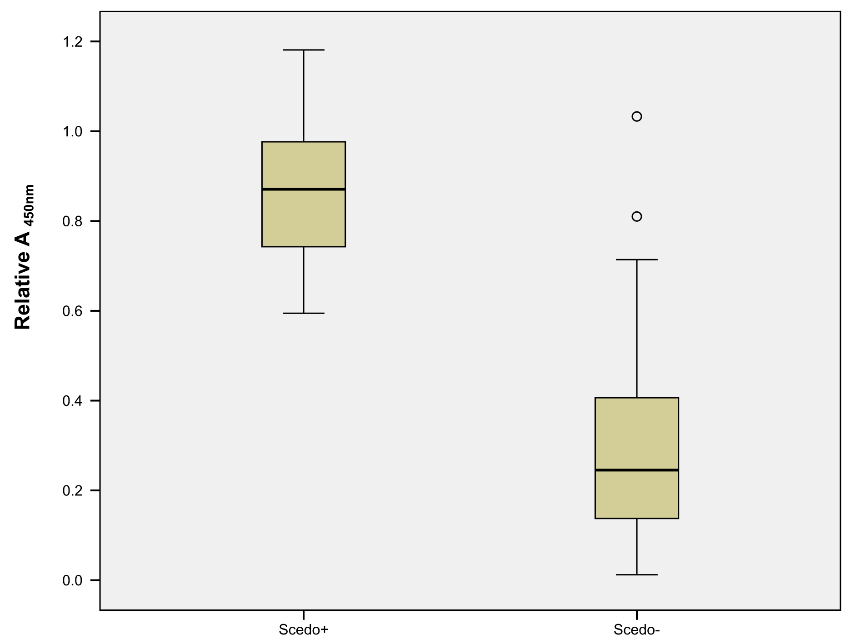
***

**Supplementary Figure 1.** Box plot of the distribution of data. Serum samples were classifies into two groups: Scedo+, sera positive for *Scedosporium / Lomentospora;* and Scedo-, rest of sera with negative culture results for these fungi.
